# 胸腔内注射重组人血管内皮抑素对裸鼠恶性胸腔积液的治疗作用

**DOI:** 10.3779/j.issn.1009-3419.2015.05.03

**Published:** 2015-05-20

**Authors:** 明 周, 敏 李, 华平 杨, 成平 胡

**Affiliations:** 410008 长沙，中南大学湘雅医院呼吸科 Department of Respiratory Medicine, Xiangya Hospital, Center South University, Hunan 410008, China

**Keywords:** 肺肿瘤, 胸腔积液, 重组人血管内皮抑素, Lung neoplasms, Pleural effusion, Recombinant human endostain

## Abstract

**背景与目的:**

恶性胸腔积液（malignant pleural effusion, MPE）临床预后不佳，胸腔内抗血管治疗可能对恶性胸腔积液具有治疗作用，本研究旨在探讨胸腔内注射重组人血管内皮抑素、顺铂、重组人血管内皮抑素联合顺铂对裸鼠恶性胸腔积液的治疗作用。

**方法:**

BALB/c裸鼠胸膜腔内注射Lewis肺癌细胞（Lewis lung cancer cell, LCC）构建恶性胸腔积液模型，造模后分别胸腔内注射重组人血管内皮抑素（E）、顺铂（P）以及重组人血管内皮抑素联合顺铂（EP）并分析各组裸鼠胸腔积液量、胸膜肿瘤微血管密度（micro vessel density, MVD）以及血管生成、凋亡相关基因的表达变化。

**结果:**

重组人血管内皮抑素及重组人血管内皮抑素联合顺铂胸腔内注射可以使裸鼠MPE量减少，且与裸鼠胸腔肿瘤组织MVD下降呈正相关；且重组人血管内皮抑素及重组人血管内皮抑素联合顺铂胸腔内注射后，MPE裸鼠胸腔肿瘤组织血管内皮生长因子（Vescular epidermal growth factor-α, VEGF-α）表达下降、低氧诱导因子-α（hypoxia induced factor-1, HIF1-α）表达升高。

**结论:**

胸腔内注射LLC细胞可成功制作裸鼠MPE模型。重组人血管内皮抑素裸鼠胸膜腔内注射对MPE裸鼠具有治疗作用，其治疗作用可能是通过下调VEGF-α，抑制肿瘤新生血管生成，下调微血管密度而达成的。

## Abstract (en)

**Background and objective:**

The prognosis of malignant pleural effusion (MPE) was poor, injecting anti-angiogenesis agents in pleural cavity might be to reducing the volume of pleural effusion. The aim of this study is to investigate the therapeutical effect of pleural injection of recombinant human endostain, cisplatin and recombinant human endostain combined with cisplatin to MPE nude mice.

**Methods:**

MPE model was built by intrpleural injection of Lewis lung cancer cells (LCC) into BALB/c nude mice. Intrpleural injection of recombinant human endostain (E), cisplatin (P) and recombinant human endostain combined with cisplatin (EP) was performed, MPE volume was measured, immunohistochemistry of CD31 was carried out to calculate micro vessel density (MVD), angiogenesis and apoptosis gene expression was detected.

**Results:**

MPE volume was reduced by intrapleiral injection of recombinant human endostain and recombinant human endostain combined with cisplatin, MPE volume was positive correlated with MVD. Vescular epidermal growth factor-α (VEGF-α) expression reduced simultaneously with expression of hypoxia induced factor-1 (HIF1-α) elevated at the same time.

**Conclusion:**

MPE model could be made by intrapleural injection of LLC. Intrapleural injection of recombinant human endostain could reduce MPE volume of nude mice. The potential molecular mechanism of the therapeutical effects of intapleural injection of recombiant endostatin might be related to the downregulation of VEGF-α expression and neovascularization.

恶性胸腔积液（malignant pleural effusion, MPE）是临床大量胸腔积液的最常见类型，约占临床所有胸腔积液的50%。恶性肿瘤对脏、壁层胸膜的侵袭和转移是MPE形成的病因机制，其中非小细胞肺癌（non-small cell lung cancer, NSCLC）为MPE的最主要病因，约15%的晚期NSCLC会发生MPE^[1]^。NSCLC-MPE患者呼吸困难症状严重，现有治疗总体疗效不佳，无系统治疗患者中位生存期仅3个月^[2]^。

目前，NSCLC-MPE治疗方法有胸腔穿刺置管、胸膜手术、胸腔镜下滑石粉胸膜内固定、胸腔内局部化疗或固定、全身化疗等^[3]^。其中，常用的胸腔内局部化疗药物有顺铂、博来霉素等^[4, 5]^。抗肿瘤新生血管药物重组人血管内皮抑素（recombinant human endostain）近年来也被作为一种胸腔内注射药物用于治疗NSCLC-MPE，但目前尚无基础及临床研究证实重组人血管内皮抑素对MPE作用机制。本研究采用经典方法构建裸鼠NSCLC-MPE模型，观察临床中常见胸腔内治疗药物顺铂及重组人血管内皮抑素对MPE的治疗作用，探讨胸腔内抗血管药物治疗的潜在机制。

## 1 材料与方法

### 1.1 实验材料

#### 1.1.1 实验动物与细胞

4周-6周雄性BALB/c裸鼠，体重18 g-22 g购买于上海斯莱克公司，饲养于中南大学实验动物学部，SPF（Specific Pathogen Free）级饲养。李维斯肺腺癌细胞系（Lewis lung cancer cell line, LLC）由中南大学肿瘤研究所教育部癌变与侵袭原理国家重点实验室提供。

#### 1.1.2 主要试剂和药品

DMEM培养基；小牛血清（Hyclone）；羊抗鼠CD31单克隆抗体艾（博抗上海贸易有限公司）；KIT-9720快捷型二抗试剂盒；NBT-2211 DAB试剂盒（福州迈新生物技术开发有限公司）；顺铂（齐鲁制药有限公司）；重组人血管内皮抑素（先声药业）；QPS-201 THUNDERBIRD SYBR qPCR Mix试剂盒（OSAKA）；RT-PCR引物[生工生物工程（上海）有限公司]。

#### 1.1.3 主要仪器

ABI 7500 PCR仪（ABI）；微量进液器50 μL、100 μL（湖南吉玛股份有限公司）；IMT-2倒置相差显微镜、OLYMPUS BX-50光学显微镜（日本Olympus公司）；计算机断层扫描（computed tomography, CT）机（Philips MX8000 EX）。

### 1.2 实验方法

#### 1.2.1 裸鼠MPE模型建立

LLC细胞系培养于37 ℃含5%CO_2_的细胞培养箱，含10%小牛血清的高糖（4.5 g/L）DMEM培养基，选择对数期肿瘤细胞，调整浓度为5×10^5^/50 μL。400 mg/kg体重剂量水合氯醛腹腔麻醉，50 μL微量进液器精确吸取50 μL细胞悬液，于BALB/c裸鼠胸骨中线胸骨右缘3 mm处垂直进针，进针深度3 mm-5 mm，推注50 μL细胞悬液。造模第13天，予以CT扫描观察，参数设置为120 Kvp，93 uA，以5 mm厚度扫描，选择造模成功的裸鼠进一步治疗。

#### 1.2.2 裸鼠胸腔内药物注射

将MPE造模成功的裸鼠20只，随机分为4组，重组人血管内皮抑素组（E组）予以胸腔内注射重组人血管内皮抑素溶液（50 μL, 25 mg/kg）^[6]^，连续给药三天；顺铂组（P组）第1天予以胸腔内注射顺铂溶液（50 μL, 25 mg/kg）^[7-9]^，第2天、第3天分别予以胸腔内注射生理盐水50 μL；联合用药组（EP组）第一天予以胸腔内注射重组人血管内皮抑素与顺铂混合溶液50 μL，第2天、第3天分别予以胸腔内注射重组人血管内皮抑素溶液（50 μL, 25 mg/kg）；对照组（NS组）连续三天予以胸腔内注射生理盐水50 μL。顺铂、重组人血管内皮抑素及混合溶液的配制均在每次给药前3 h内完成。

#### 1.2.3 标本的处理

MPE裸鼠在完成胸腔内药物注射24 h后，采用眼底静脉放血法处死裸鼠，微量进液器逐一吸取胸腔积液，确定胸水量。剪下胸壁胸膜上肿瘤组织，一份组织于4%中性福尔马林中固定，另一份-20 ℃冰箱冻存备用。

#### 1.2.4 免疫组化分析各组胸腔肿瘤MVD

将组织切片，按照以下步骤进行免疫组化分析：烤片，脱蜡，水化，抗原修复，以1:200滴加一抗，加二抗、DAB染色等。

#### 1.2.5 RT-PCR分析各组胸腔肿瘤中血管内皮生长因子（Vescular epidermal growth factor-α, VEGF-α）、低氧诱导因子-α（hypoxia induced factor-1, HIF1-α）、*Bax*及*Bcl-2*基因的表达

利用Beacon designer 7.9设计*VEGF-α*、*HIF1-α*、*Bax*及*Bcl-2*基因引物，并验证上述基因引物，上海生工合成；引物为VEGF-α（ACACCCACCCACATACACAC, GCCTTTCATCCCATTGTCTC），HIF1-α（CAAGCCCTCCAAGTATGAGC, ATGCCTTAGCAGTGGTCGTT），Bax（ATGCGTCCACCAAGAAGC, GCAAAGTAGAAGAGGGCAACC），Bcl-2（CGATTGTGGCAGTCCCTTA, CCAGGATGAAGTGCTCAGGT）内参为GAPDH（GCAGTGGCAAAGTGGAGATT, CCTTGACTGTGCCGTTGAAT），扩增长度114 bp。

按照10 μL反应逆转录体系配置反应溶液，上机逆转录，设置反应条件：①37 ℃ 15 s；②37 ℃ 15 min；③4 ℃ 15 min后终止逆转录。20 μL体系配置PCR反应液，20 μL体系中，SYBR qPCR Mix 10 μL，上下游引物Mix 1.2 μL（6 pmol），50X ROX reference 0.4 μL（ABI 7500RT-PCR仪器用0.4 μL），cDNA 4 μL，灭菌蒸馏水4.4 μL补齐体系；设置参数，95 ℃ 60 s预变性，（95 ℃ 15 s PCR变性，60 ℃ 60 s PCR反应）^*^40 cycle；分析PCR数据结果。RT-PCR数据结果，采用经典ΔΔCT的分析方法，进行组间基因表达差异分析。

#### 1.2.6 统计学分析

统计软件为SPSS 19.0。所有实验数据采用均数±标准差（Mean±SD）表示，组间均数的比较采用多样本均数比较和方差分析，*P*值均为双侧概率，检验水准α=0.05，*P* < 0.05为差异有统计学意义。

## 2 结果

### 2.1 MPE造模情况

裸鼠MPE造模第13天，55%（22/40）的裸鼠出现气促、胸廓饱满，同时伴有活动、饮水、进食量减少（图 1）。其中50%（20/40）的裸鼠符合以下证据，提示MPE造模成功^[6, 10]^：①裸鼠CT示存在胸腔积液。②裸鼠解剖观察胸腔内存在血性、不凝固液体。③裸鼠胸膜或肺表面可观察到结节病灶。④通过病理分析可确定结节为肿瘤。依照上述因素，造模成功率为50%。

**1:**
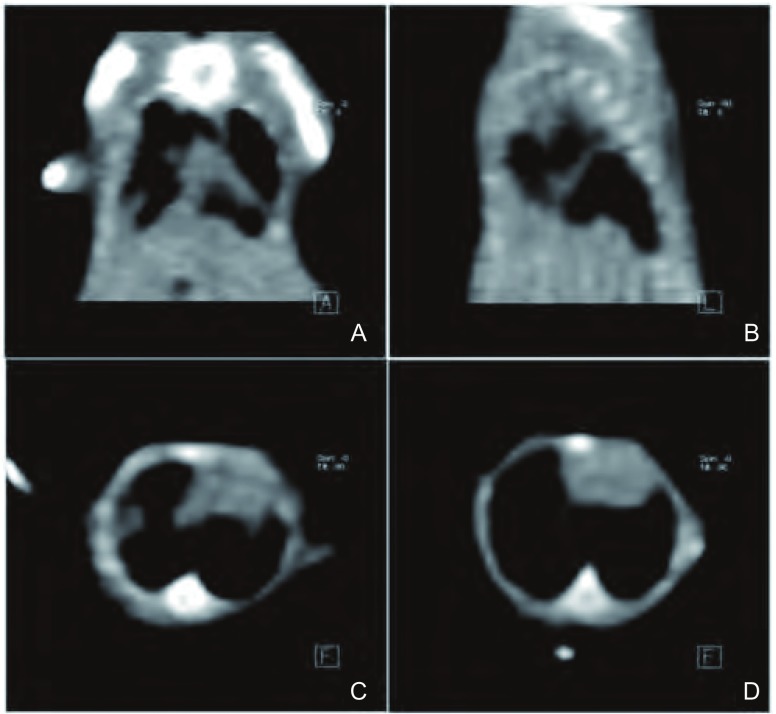
MPE造模第13天裸鼠CT扫描图。A：裸鼠成功造模后，冠状面CT扫描图；B：该裸鼠的矢状面CT扫描图；C：胸骨中段横断面CT扫描图，可见肋膈角变钝，提示存在胸腔积液；D：该裸鼠接受重组人血管内皮抑素治疗三天后同一层面的横断面CT扫描图，可见胸腔积液减少 CT scan results of MPE nude mice at the 13th day after pleural injection. A: After MPE nude mice model was made, coronal plane CT scan result; B: Vertical plane CT scan result; C: Cross-section CT scan of sternum, blunting costophrenic angle suggested MPE model was made; D: Cross-section CTscan of sternum of the same nude mice after 3-day recombinant human endostain treatment, which showed MPE volume was reduced. MPE: malignant pleural effusion; CT: computed tomography

### 2.2 胸腔内药物注射对MPE量的影响

各组裸鼠MPE量的均值和标准差分别为：E组（271.8±133.8）μL；P组（447.4±75.3）μL；EP组（247.2±89.2）μL；NS组（503.2±146.6）μL，对比NS组，各治疗组MPE量不同程度减少（图 2）。对比NS组，EP组MPE量减少，差异具有统计学意义（*P*=0.042, 8）；E组及P组MPE量也减少，差异无统计学意义。对比P组，E组、EP组MPE量也减少，差异无统计学意义。对比E组，EP组MPE量略减少，差异无统计学意义。在正式试验造模及给药中，3/40存在少量穿刺点出血的不良事件，无气胸、死亡等严重不良事件发生。

**2:**
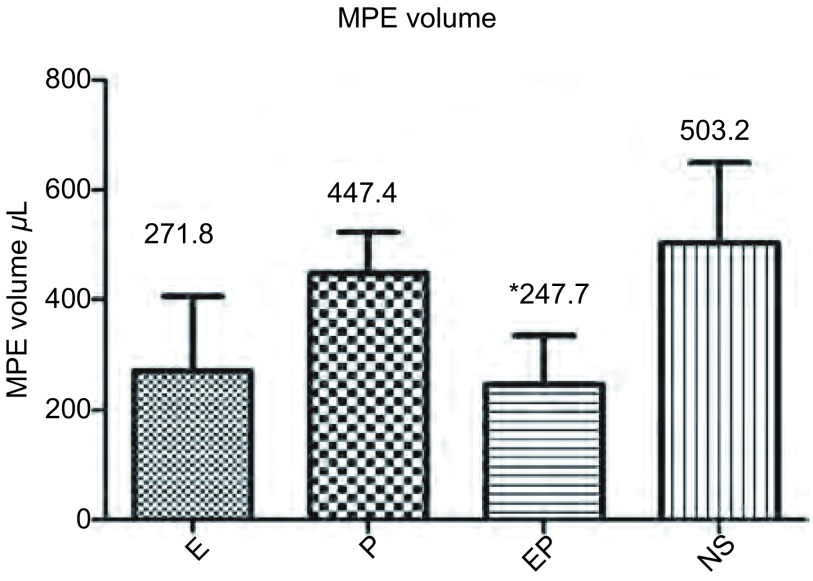
MPE裸鼠胸腔内药物注射后的胸水量。柱状分析图显示重组人血管内皮抑素及重组人血管内皮抑素联合顺铂可使胸腔积液量减少。^*^：与NS相比，*P* < 0.05。E：重组人血管内皮抑素；P：顺铂；EP：重组人血管内皮抑素+顺铂；NS：生理盐水 MPE volume of MPE nude mice after intrapleural injection. The result reveals that recombinant human endostain and recombinant human endostain combined with cisplatin could reduce MPE volume. ^*^: Compared with NS group, *P* < 0.05. E: recombinant human endostain; P: cisplatin; EP: recombinant human endostain+cisplatin; NS: normal saline

### 2.3 MPE裸鼠胸腔肿瘤的MVD分析

MVD的判断：任何被抗体染色的单个细胞或细胞团，不管它是否形成管腔，只要它与周围的血管、肿瘤细胞和其他连接组织成分有一个清楚的分隔，都认为是一个可计数的微血管。肿瘤内硬化区以及与肿瘤交界处软组织内的微血管不计数，有厚的平滑肌壁或管腔直径>8个红细胞直径的血管也排除在外。对各组裸鼠的胸腔肿瘤组织进行CD31免疫组化后，可见E组、EP组较NS组的CD31深染管腔及裂隙明显减少，具体如图 3。

**3:**
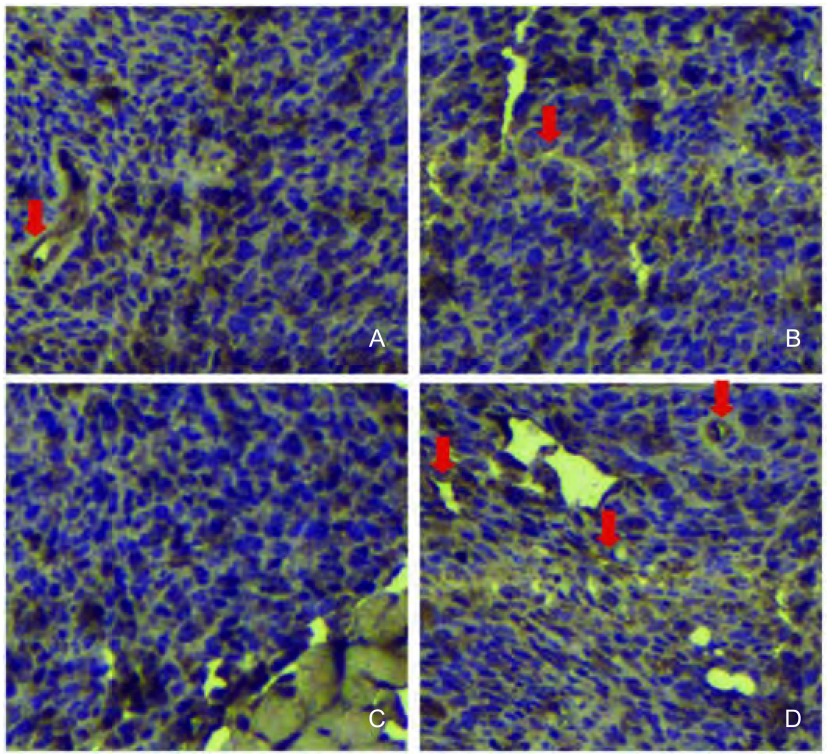
MPE裸鼠胸腔肿瘤的CD31免疫组化染色（×200）。A：重组人血管内皮抑素治疗组MPE裸鼠肿瘤组织CD31免疫组化染色，对比对照组，重组人血管内皮抑素治疗后MVD下降；B：对比对照组，顺铂治疗后MVD未下降；C：对比对照组，重组人血管内皮抑素联合顺铂治疗后MVD下降；D：生理盐水空白对照组；MVD：微血管密度 CD31 immunohistochemistry of tumor tissues of MPE nude mice (×200). A: Immunohistochemistry for CD31 of recombinant human endostain treatment group, MVD decreased after recombinant human endostain treatment; B: Cisplatin group, MVD did not decrease compared with control group; C: Recombinant human endostain combined with cisplatin group, MVD decreased after combined treatment; D: Control group treated with normal saline. MVD: microvessel density

对每组每只MPE裸鼠随机取切片中3个高倍镜（×200）视野，计数MVD。经过计算，各组MVD均值及标标准差分别为：E组12.34±2.21；P组26.54±4.23；EP组11.64±2.24；NS组28.42±3.30（图 4）。

**4:**
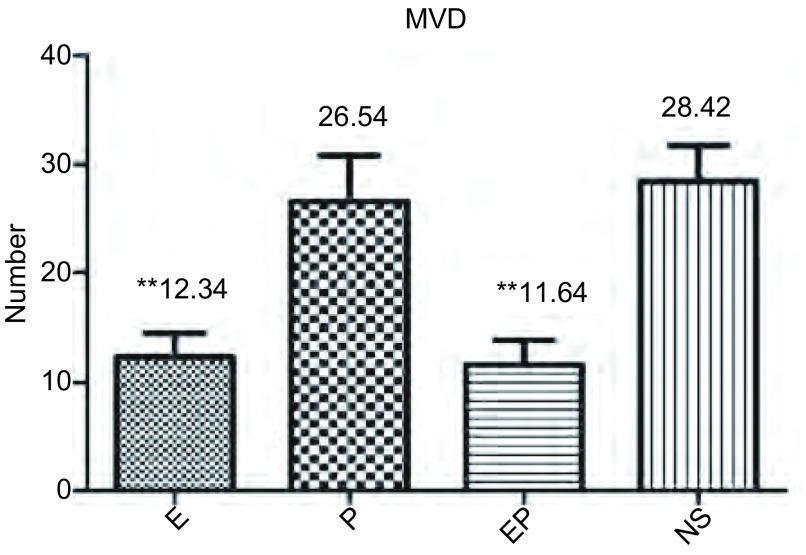
MPE裸鼠胸腔肿瘤的微血管密度。柱状分析图显示重组人血管内皮抑素及重组人血管内皮抑素联合顺铂组肿瘤微血管密度减少。^**^：与NS相比，*P* < 0.01 Mean blood vessel density of MPE nude mice tumor tissues. The result reveals that recombinant human endostain and recombinant human endostain combined with cisplatin could reduce mean blood vessel density. ^**^: Compared with NS, *P* < 0.01

对比NS组，E组、EP组MVD减少，差别有统计学意义（*P*=0.000, 8, *P*=0.000, 7），P组MVD略减少，差别无统计学意义。各治疗组间，对比P组，E组、EP组MVD明显下降，差别有统计学意义（*P*=0.002, 4, *P*=0.003, 0）；EP组和E组间差别无统计学意义。

以MPE裸鼠胸腔内药物注射后的胸水量为纵坐标、MPE裸鼠胸腔肿瘤的MVD为横坐标做散点图（图 5），拟合后可观察到MPE量与MVD大致成正相关。该线性回归模型相关系数R为0.714493≈0.71，决定系数R^2^=0.510, 5，对回归模型进行*F*检验，回归方程有统计学意义（*F*=18.77, *P*=0.000, 4）。

**5:**
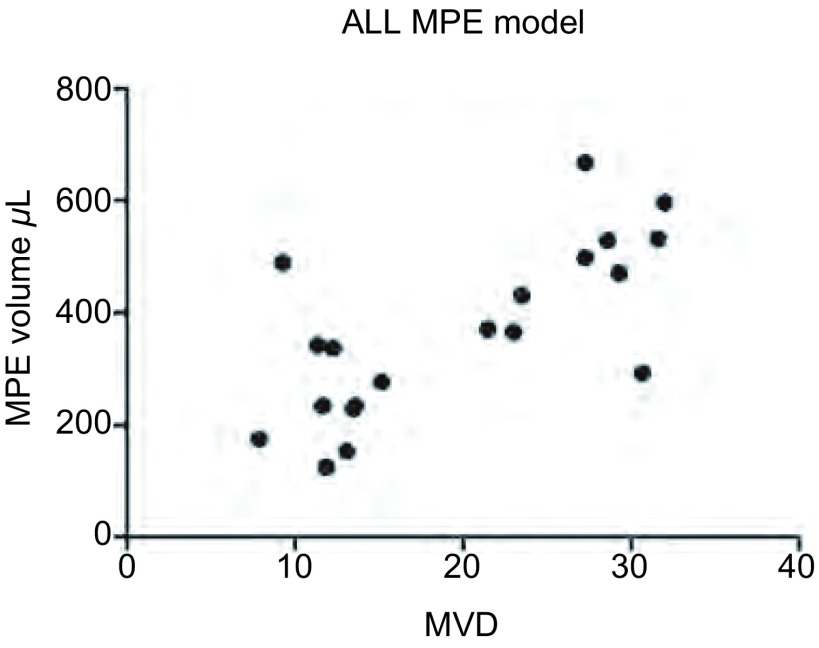
MPE量与MVD关系散点图。散点图提示，MPE量与MVD大致成正相关 Scatter plot of MVD and MPE volume. Scatter plot suggested MPE volume was positive correlation with MVD

### 2.4 MPE裸鼠胸腔肿瘤血管生成及凋亡相关基因表达分析

本实验选择了与血管生成密切相关的基因VEGF-α和HIF1-α以及与细胞凋亡密切相关的基因Bax和Bcl-2进行RT-PCR分析。在血管生成相关基因的表达分析中，抗血管治疗后，VEGF-α表达下降，而HIF1-α表达升高。对比NS组，E组及EP组VEGF-α的表达下降，差别具有统计学意义（均*P* < 0.000, 1），而E组及EP组HIF1-α的表达升高，差别具有统计学意义（*P*=0.031, 2, *P*=0.000, 2）；凋亡相关基因显示，顺铂治疗后*Bax*基因的表达升高，差别具有统计学意义（均*P*=0.000, 3），但Bcl-2的表达差异无统计学意义（图 6）。

**6:**
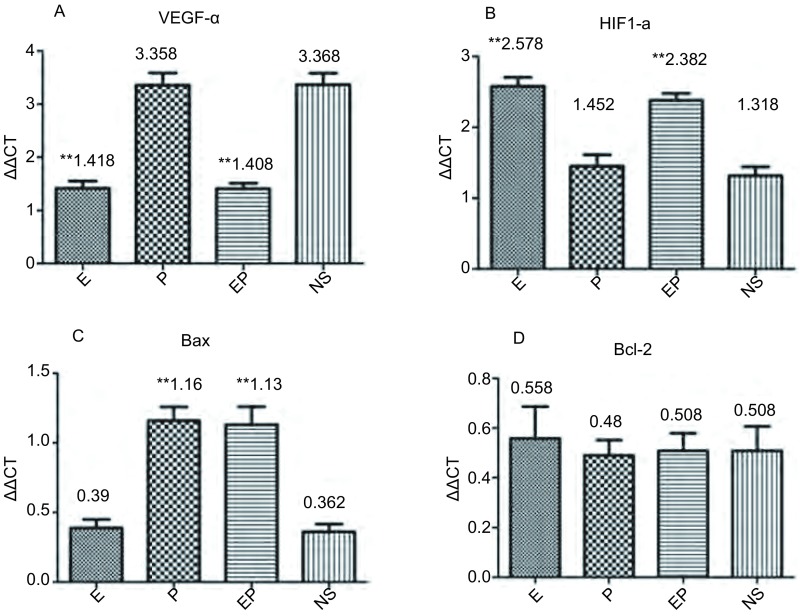
MPE裸鼠胸腔肿瘤的基因表达分析。A：VEGF-α表达分析提示，重组人血管内皮抑素治疗后VEGF-α表达下降；B：HIF1-α表达分析提示，重组人血管内皮抑素治疗后HIF1-α表达升高；C：Bax表达分析提示，顺铂治疗后Bax表达升高；D：Bcl-2表达分析，各组治疗后Bcl-2表达无差异。^**^：与NS相比，*P* < 0.01 Gene expression analysis of MPE nude mice tumor tissues. A: The expressions of VEGF-*α*, suggested that expression of VEGF-*α* decreased after recombinant human endostain treatment; B: The expressions of HIF1-*α*, suggested that expression of HIF1-*α* elevated after recombinant human endostain treatment; C: The expression of Bax, suggested that the expression of Bax elevated after cisplatin treatment; D: The expression of Bcl-2, suggested that the expression of Bcl-2 did not change after treatment. ^**^: Compared with NS, *P* < 0.01. VEGF-*α*: Vescular epidermal growth factor-*α*, VEGF-*α*; HIF1-*α*: hypoxia induced factor-1

## 3 讨论

晚期肺癌合并MPE的治疗经历了胸腔穿刺、局部胸膜切除手术治疗、胸腔镜下滑石粉胸膜内固定，到全身联合化疗、胸腔内局部化疗等过程。而近年来肺癌MPE治疗进展则主要集中在胸腔内药物治疗领域。

本实验采用经典的胸腔内注射LLC细胞造模方法，并以CT影像学、大体解剖以及病理学方法确定成功构建MPE裸鼠模型。尽管临床中使用顺铂胸腔内注射治疗NSCLC-MPE较为常见，但本研究中顺铂对裸鼠MPE的治疗效果并不明显；而重组人血管内皮抑素及重组人血管内皮抑素联合顺铂胸腔内注射却显示出对裸鼠MPE具有一定的治疗作用。重组人血管内皮抑素及重组人血管内皮抑素联合顺铂胸腔内注射可以使裸鼠MPE量减少，且与裸鼠胸腔肿瘤组织MVD下降呈正相关。这提示重组人血管内皮抑素对裸鼠MPE的治疗作用可能是通过其抑制肿瘤新生血管生成，下调微血管密度而达成的。

近年，针对MPE发生机制的研究主要集中于肿瘤侵袭、代谢、免疫及血管生成等。肿瘤血管生成活跃，新生血管管腔不完整，管壁通透性高，有利于炎症细胞和大分子蛋白透过，血管因素可能为MPE形成的核心机制。临床研究提示，恶性胸腔积液中VEGF-α、IL-8以及血管生成素水平升高，其中VEGF-α水平和胸腔积液量正相关^[11]^；针对VEGFR的治疗可减少MPE的形成^[12]^。Yeh等^[13]^发现，在肺腺癌中，自分泌IL-6/STAT3/VEGF的途径可能通过促进新生血管生成促进MPE。2009年，Fang等^[14]^的研究提示，用表达重组人血管内皮抑素的腺病毒治疗MPE小鼠可降低胸腔积液量及肿瘤微血管密度，和胸腔内注射腺病毒相比，重组人血管内皮抑素更加安全，本研究发现重组人血管内皮抑素胸腔注射后，MPE裸鼠胸腔肿瘤组织VEGF-α表达下降，提示其抗MPE生成作用可能是通过下调VEGF-α来实现的。MPE裸鼠胸腔肿瘤组织HIF1-α表达在重组人血管内皮抑素胸腔注射后升高，提示肿瘤局部缺氧，抗血管治疗有效；但有趣的是低氧导致HIF1-α的激活，活化的HIF1-α入核作为一种转录因子，又可激活血管生长因子，为肿瘤血管生成始动因素^[15]^，因此重组人血管内皮抑素胸腔注射对MPE裸鼠胸腔肿瘤组织HIF1-α表达升高，到底孰因孰果还有待进一步研究探明。

本研究中对MPE裸鼠胸腔肿瘤组织Bax、Bcl-2的表达分析提示，顺铂胸腔内注射虽然有促进裸鼠胸腔肿瘤凋亡的趋势，但顺铂的促凋亡作用却并未转化为对MPE的治疗优势，这有可能是因为临床实践中胸腔内注射顺铂的主要作用是顺铂引起的胸膜腔无菌性炎症，而非顺铂的抗肿瘤作用；且在临床操作中，利用顺铂做胸腔内固定治疗前，需要将胸水引流完全，而目前仍没有在不处死裸鼠的情况下将裸鼠胸腔内积液精确引流的实验方法。因此，本研究结果并不能否定顺铂胸腔内注射对MPE的治疗效果，而如何创造条件实现裸鼠胸腔积液在体引流将有待进一步研究探讨。
